# Possible activation by the green tea amino acid theanine of mammalian target of rapamycin signaling in undifferentiated neural progenitor cells *in vitro*

**DOI:** 10.1016/j.bbrep.2015.09.021

**Published:** 2015-12-01

**Authors:** Takeshi Takarada, Noritaka Nakamichi, Ryota Nakazato, Takami Kakuda, Hiroshi Kokubo, Shinsuke Ikeno, Saki Nakamura, Nobuyuki Kuramoto, Eiichi Hinoi, Yukio Yoneda

**Affiliations:** a Laboratory of Molecular Pharmacology, Division of Pharmaceutical Sciences, Kanazawa University Graduate School of Medical, Pharmaceutical and Health Sciences, Kanazawa 920-1192, Japan; b Research Center of Composite Material, Fukuoka University, Fukuoka 814-0180, Japan; c Department of Toxicology, Faculty of Pharmaceutical Sciences, Setsunan University, Hirakata 573-0101, Japan

**Keywords:** Theanine, Neural progenitors, mTOR, SLC38A1, Neurogenesis, Glutamine

## Abstract

We have shown marked promotion of both proliferation and neuronal differentiation in pluripotent P19 cells exposed to the green tea amino acid theanine, which is a good substrate for SLC38A1 responsible for glutamine transport. In this study, we evaluated the activity of the mammalian target of rapamycin (mTOR) kinase pathway, which participates in protein translation, cell growth and autophagy in a manner relevant to intracellular glutamine levels, in murine neural progenitor cells exposed to theanine. Exposure to theanine promoted the phosphorylation of mTOR and downstream proteins in neurospheres from embryonic mouse neocortex. Although stable overexpression of SLC38A1 similarly facilitated phosphorylation of mTOR-relevant proteins in undifferentiated P19 cells, theanine failed to additionally accelerate the increased phosphorylation in these stable transfectants. Theanine accelerated the formation of neurospheres from murine embryonic neocortex and adult hippocampus, along with facilitation of both 5-bromo-2’-deoxyuridine incorporation and 3-(4,5-dimethyl-2-thiazolyl)-2,5-diphenyl-2H-tetrazolium bromide reduction in embryonic neurospheres. In embryonic neurospheres previously exposed to theanine, a significant increase was seen in the number of cells immunoreactive for a neuronal marker protein after spontaneous differentiation. These results suggest that theanine activates the mTOR signaling pathway for proliferation together with accelerated neurogenesis in murine undifferentiated neural progenitor cells.

## Highlights

• Theanine promotes mTOR phosphorylation in neural progenitors.
• Theanine promotes the formation of embryonic and adult neurospheres.
• SLC38A1 overexpression promotes mTOR phosphorylation in pluripotent P19 cells.
• Theanine fails to further promote mTOR phosphorylation in SLC38A1 transfectants.
• Theanine promotes subsequent neuronal differentiation in embryonic progenitors.

## 1 Introduction

Theanine (=γ-glutamylethylamide) is an amino acid ingredient in green tea with structural analogy to glutamine (Gln), which is a precursor for amino acid neurotransmitters such as γ-aminobutyric and glutamic acids, in the brain [1]. Although an intracerebroventricular injection of theanine protected hippocampal CA1 pyramidal neurons from delayed neuronal death in brains of gerbils with bilateral forebrain global ischemia *in vivo* [2], theanine was a poor inhibitor of ligand binding to different ionotropic glutamate receptor subtypes with high neurotoxicity in rat cortical synaptic membranes *in vitro* [3]. Theanine inhibited [^3^H]Gln incorporation without affecting [^3^H]glutamate uptake in rat brain synaptosomes, while both [^3^H]theanine and [^3^H]Gln were similarly taken up in a sodium- and structure-dependent manner [4]. Theanine intake was shown to promote the object recognition memory, along with an increase in 5-bromo-2′-deoxyuridine (BrdU) incorporation into the hippocampus, in developing young rats [5]. Similarly, theanine prevented the cognitive dysfunction in accelerated-senescence mice *in vivo* [6]. We have recently shown that daily oral intake of theanine alleviated a variety of behavioral abnormalities, in addition to preventing a transient decline of BrdU incorporation into the hippocampal dentate gyrus, in adult mice with traumatic severe stress [7].

In contrast, intracellular Gln was shown to participate in activation of the mammalian target of rapamycin (mTOR) kinase signaling system responsible for protein translation, cell growth and autophagy through a mechanism relevant to several membrane transporters for Gln in cultured HeLa cells [8]. Evidence for the mTOR pathway as an intracellular downstream signal of extracellular essential amino acids is now accumulating [9]. Marked promotion of cellular proliferation and neuronal differentiation was seen in pluripotent P19 cells with stable overexpression of solute carrier 38a1 (SLC38A1=glutamine transporter, GlnT), which is responsible for the membrane transport of Gln in the brain [10]. Theanine similarly accelerated both activities in control stable transfectants with *empty vector* (EV), but failed to further facilitate the promotion of both proliferation and neuronal differentiation activities in stable *Slc38a1* transfectants [11].

These previous findings prompted us to investigate the activity of mTOR signaling-related molecules in neural progenitor cells exposed to theanine. For this purpose, we employed in this study neural progenitor cells isolated from embryonic mouse neocortex enriched of primitive cells immunoreactive for the undifferentiated progenitor cell marker nestin [12], in addition to the murine embryonal carcinoma cell line P19 cells with pluripotency. We also evaluated pharmacological actions of theanine on the proliferation in hippocampal progenitor cells [13] isolated from adult mice with predominant overexpression of green fluorescent protein (GFP) in cells expressing nestin [14].

## 2 Methods

### 2.1 Materials

Materials used were obtained from different sources described below. Theanine, Tokyo Kasei Kogyo (Tokyo, Japan); Pluripotent P19 stem cells derived from murine embryonal carcinoma, Riken Cell Bank (Tsukuba, Japan); Antibodies against microtubules-associated protein-2 (MAP2) and glial fibrillary acidic protein (GFAP), 3-(4,5-dimethyl-2-thiazolyl)-2,5-diphenyl-2H-tetrazolium bromide (MTT), ciliary neurotrophic factor (CNTF) and all-*trans* retinoic acid (ATRA), Sigma Chemicals (St. Louis, MO, USA); Antibody against BrdU, Abcam (Cambridge, UK); Antibodies for phosphorylated mTOR, p70 S6 kinase (p70S6K), phosphorylated-p70S6K and phosphorylated-S6, Cell Signaling Technology (Danvers, MA, USA); Anti-rabbit IgG antibody conjugated with peroxidase and ECL™ detection reagent, Amersham (Buckinghamshire, UK); Dulbecco’s modified Eagle medium (DMEM), DMEM: Nutrient Mixture F-12 (DMEM/F-12) 1:1 Mixture, alpha minimal essential medium (αMEM), StemPro Accutase, GlutaMAX, B27 supplement and fetal bovine serum (FBS), GIBCO BRL (Gaithersburg, MD, USA), Epidermal growth factor (EGF) and fibroblast growth factor (FGF), Biomedical Technologies (Stoughton, MA, USA).

### 2.2 Animals use

The protocol employed here meets the guideline of the Japanese Society for Pharmacology and was approved by the Committee for Ethical Use of Experimental Animals at Kanazawa University (Permit no. 70093). All efforts were invariably made to minimize animal suffering, to reduce the number of animals used and to utilize alternatives to *in vivo* techniques.

### 2.3 Embryonic mouse neural progenitor cells

Neocortex was isolated from 15.5-day-old embryonic Std-ddY mice (Japan SLC, Inc., Shizuoka, Japan), followed by trituration through a Pasteur pipette with enzyme cocktails in phosphate-buffered saline (PBS) and subsequent collection of the lower layer enriched of undifferentiated progenitor cells after Percoll centrifugation procedures [13], [15]. Cells were cultured for 12 days under floating conditions at 37 °C under 5% CO_2_ in a humidified CO_2_ incubator with a half medium change every 2 days.

### 2.4 Proliferation

Cultured neural progenitor cells were exposed to theanine for 12 days in the presence of EGF. Proliferation activity was then quantified by MTT reduction assays [10]. Neurospheres cultured with theanine for 8 days were incubated with 10 μM BrdU for 10 h, followed by culture for 2 h and subsequent fixation with 4% paraformaldehyde (PA) for the immunocytochemical detection of BrdU [12]. Neurospheres were cultured with theanine for 6 days, followed by further culture for 2 h and fixation with 4% PA for double staining for DNA with 10 μg/ml Hoechst33342 and 5 μg/ml propidium iodide (PI) [12].

### 2.5 Differentiation

The cells were dissociated by pipetting, followed by seeding at 15,000 cells/well and subsequent culture without theanine for an additional 6 days [10]. Cells were then subjected to immunocytochemistry with antibodies against MAP2 (1:500) and GFAP (1:500) overnight at 4 °C. Quantification was performed by counting the number of cells immunoreactive for either MAP2 or GFAP in a blinded fashion, followed by calculation of the individual percentages over the number of total cells stained with Hoechst33342 [12]. For Western blotting, cells were subjected to sodium dodecyl sulfate polyacrylamide gel electrophoresis [11] using antibodies against phospho-p70 S6 kinase (1:1,000), p70 S6 kinase (1:1,000), phosphor-S6 (1:1,000) and glyceraldehyde-3-phosphate dehydrogenase (GAPDH) (1:4,000). The relative amount of each protein was normalized by the quantitative densitometric analysis using Image J software.

### 2.6 Stable Slc38a1 transfectants

Mouse embryonal carcinoma P19 cells were plated at a density of 1.5×10^5^ cells/cm^2^, followed by culture in DMEM with 10% FBS for 24 h and subsequent stable transfection with pSI- GlnT, pSI-GFP and pcDNA3.1 vectors, or pSI, pSI-GFP and pcDNA3.1 vectors, using 2 µg of DNA and Lipofectamine and Plus reagent in 0.5 ml of Opti-MEM. Cells were selected after culture in medium containing 500 µg/mL of G418 for establishment of stable transfectants with *Slc38a1* expression vector and *EV* in P19 cells for further studies using clones between passages 3–6 as described previously [10].

### 2.7 Adult mouse neural progenitors

Hippocampus was isolated from 4-week-old *Nestin*-GFP transgenic mice, which were kindly provided by Dr. Grigori Enikolopov (Cold Spring Harbor Laboratory, NY, USA) [14], followed by mechanical trituration and culture in DMEM/F12 containing 1xGlutaMAX, 1xB27 supplement, 20 ng/mL EGF and 20 ng/mL FGF for 14 days under floating conditions. Resultant neurospheres were dissociated by StemPro Accutase, followed by culture at 300 cells/well with theanine in DMEM/F12 containing 1xGlutaMAX, 1xB27 supplement, 20 ng/mL EGF and 20 ng/mL FGF for an additional 24 days under floating conditions for the analysis of neurosphere size. Five different visual fields were chosen at random from each culture well under a phase contrast micrograph in a blinded fashion, followed by calculation of the size of neurospheres composed of clustered proliferating cells in parallel experiments for summation using the Scion Image β 4.02 software as described previously [12].

### 2.8 Data analysis

All results are expressed as the mean±SE, and the statistical significance was determined by the one-way or two-way ANOVA with Bonferroni/Dunnett’s *post hoc* test. The level of significance was set at *p*<0.05.

## 3 Results

### 3.1 mTOR signaling pathway

**Fig. 1:**
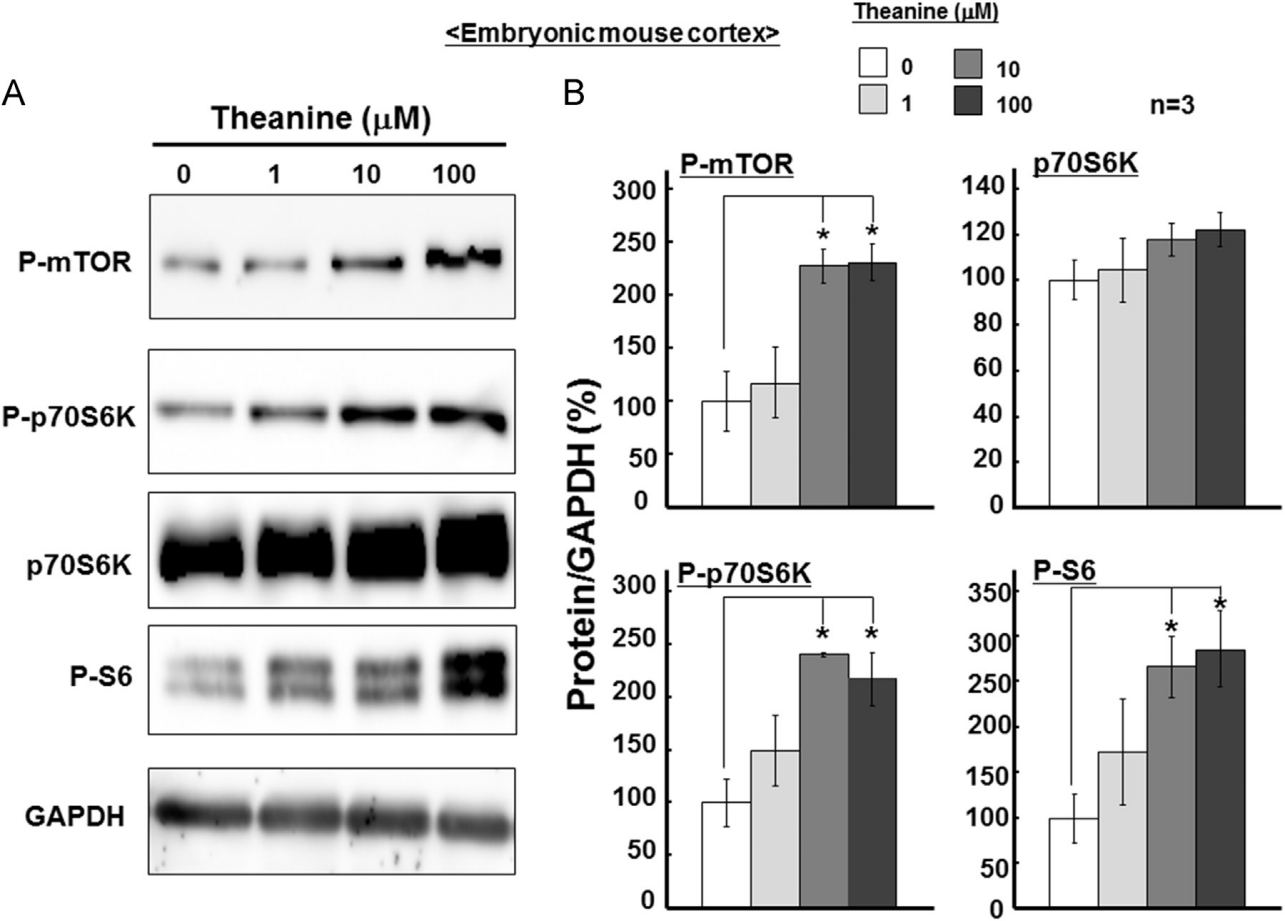
Effects of theanine on mTOR signaling molecules in murine neural progenitor cells. Neural progenitor cells from embryonic mouse neocortex were cultured with EGF in either the presence or absence of theanine at different concentrations for 10 days, followed by measurement of endogenous levels of particular proteins required for the mTOR signaling on Western blotting. Typical pictures are shown in the panel (A), while the panel (B) shows quantitative data of each protein level (*n=*3). *P<0.05, significantly different from each control value obtained in cells not exposed to theanine. Statistical significance was determined using the one-way ANOVA with Bonferroni/Dunnett *post hoc* test.

We at first investigated the phosphorylation of several key proteins related to the mTOR kinase pathway shown to be responsive to different amino acid signals [8], [9] in undifferentiated murine neocortical neurospheres cultured with theanine for 10 days. Theanine at 1–100 μM drastically accelerated the phosphorylation of both mTOR and its substrate, p70S6K, without markedly affecting p70S6K levels (Fig. 1A). Subsequent quantification confirmed statistically significant promotion of the phosphorylation of mTOR, p70S6K and S6, without significantly altering p70S6K levels, in murine neurospheres cultured with theanine at 10–100 μM for 10 days (Fig. 1B).

### 3.2 Theanine in stable Slc38a1 transfectants of P19 cells

**Fig. 2:**
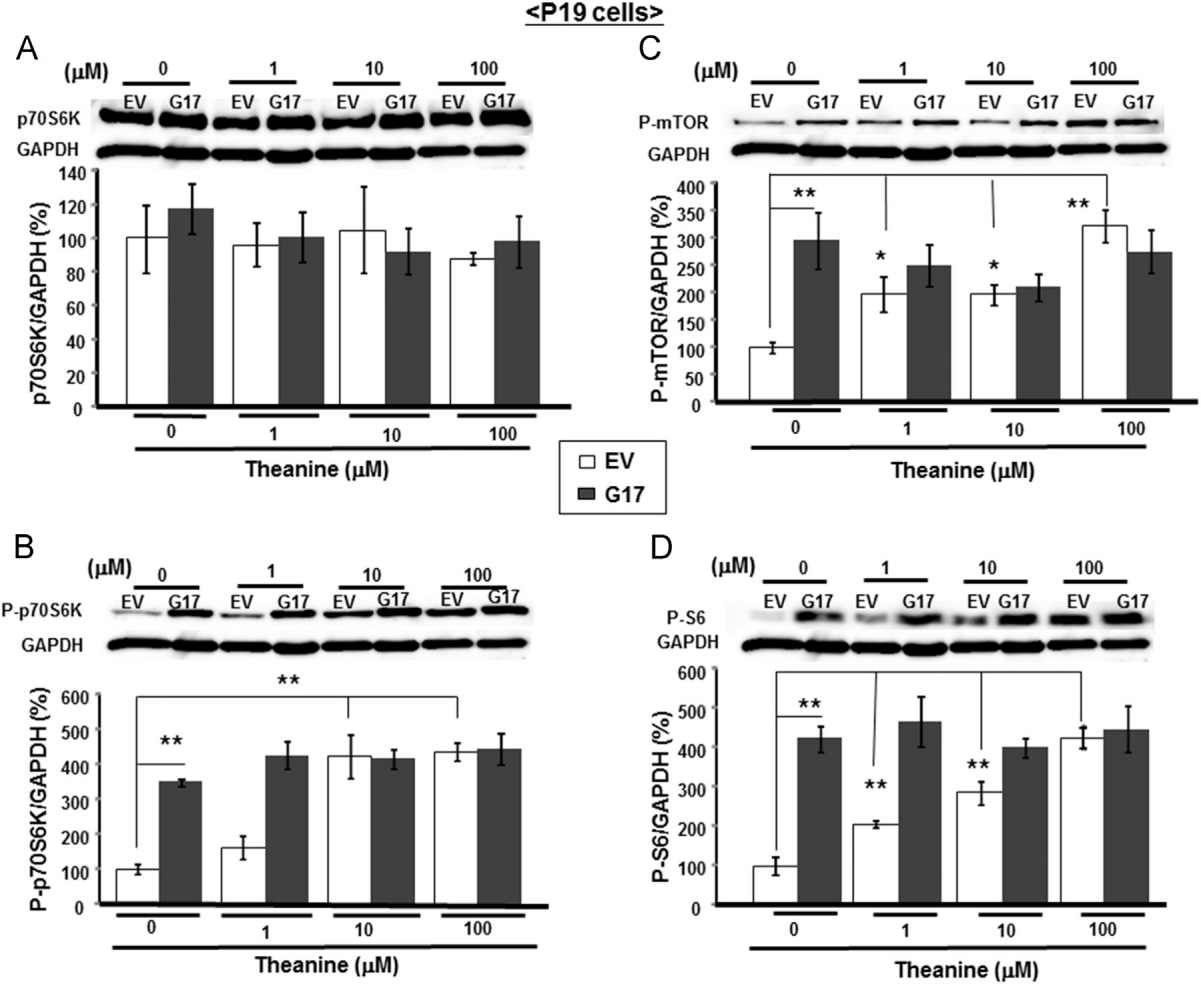
Effects of theanine on mTOR signaling molecules in stable *Slc38a1* transfectants in P19 cells. Undifferentiated stable transfectants with *EV* or *Slc38a1* were cultured with 0.5 μM ATRA in either the presence or absence of theanine at different concentrations for 4 days, followed by measurement of endogenous levels of (A) p70S6K, (B) P-p70S6K, (C) P-mTOR and (D) P-S6 on Western blotting. Typical gel pictures are shown in the upper lanes of each panel, while the lower columns show quantitative data of each protein level (*n*=3). *P<0.05, **P<0.01, significantly different from each control value obtained in cells not exposed to theanine. Statistical significance was determined using the two-way ANOVA with Bonferroni/Dunnett *post hoc* test.

We evaluated the phosphorylation of mTOR-relevant proteins in P19 cells stably overexpressing SLC38A1 which is capable of transporting both Gln and theanine in the brain. Stable overexpression of *Slc38a1* did not markedly affect the endogenous level of p70S6K in the absence of theanine (Fig. 2A), but drastically promoted the phosphorylation of p70S6K (Fig. 2B), mTOR (Fig. 2C) and S6 (Fig. 2D). The addition of theanine led to significant acceleration of the phosphorylation of p70S6K, mTOR and S6 in a concentration-dependent manner in control cells with *EV* as seen in neural progenitor cells, but failed to additionally promote the phosphorylation of these intracellular molecules relevant to the mTOR signaling even at the highest concentration used in stable *Slc38a1* transfectants. In stable *Slc38a1* transfectants, theanine was ineffective in affecting the endogenous level of p70S6K irrespective of the concentrations employed.

**Fig. 3:**
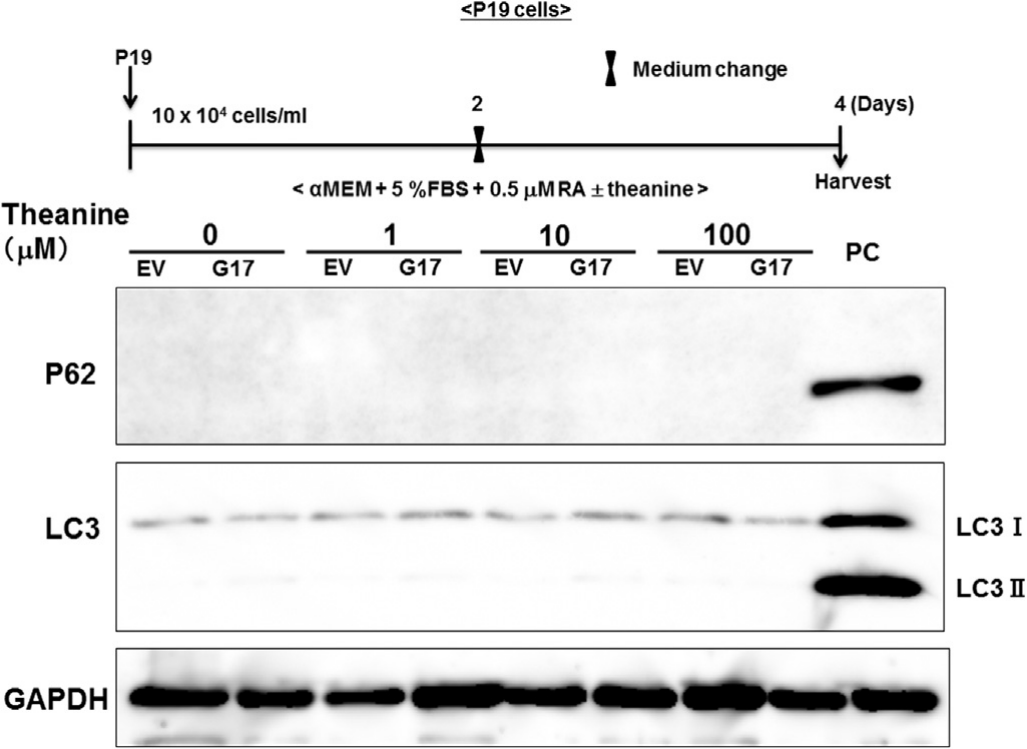
Effects of theanine of MAP1LC3 levels in stable P19 transfectants. Stable transfectants with *EV* or *Slc38a1* were cultured with ATRA in either the presence or absence of theanine at different concentrations for 4 days, followed by measurement of endogenous levels of MAP1LC3-I and MAP1LC3-II on Western blotting. PC: positive control, Neuro2a cells.

Since evidence is accumulating for involvement of the mTOR signaling pathway in mechanisms underlying prevention of eukaryotic cells from undergoing autophagy [16], an attempt was made to determine whether theanine modulates the activity of autophagy in association with promotion of the phosphorylation of mTOR signaling molecules in P19 cells. Western blotting analysis revealed considerably low endogenous levels of the autophagy marker microtubule-associated protein-1 light chain-3 (MAP1LC3) in P19 cells, however, whereas endogenous levels of both MAP1LC3-I and MAP1LC3-II were not markedly affected in cells with sustained exposure to theanine at 1–100 μM for 4 days irrespective of stable overexpression of SLC38A1 (Fig. 3).

### 3.3 Theanine in mouse neural progenitors

**Fig. 4:**
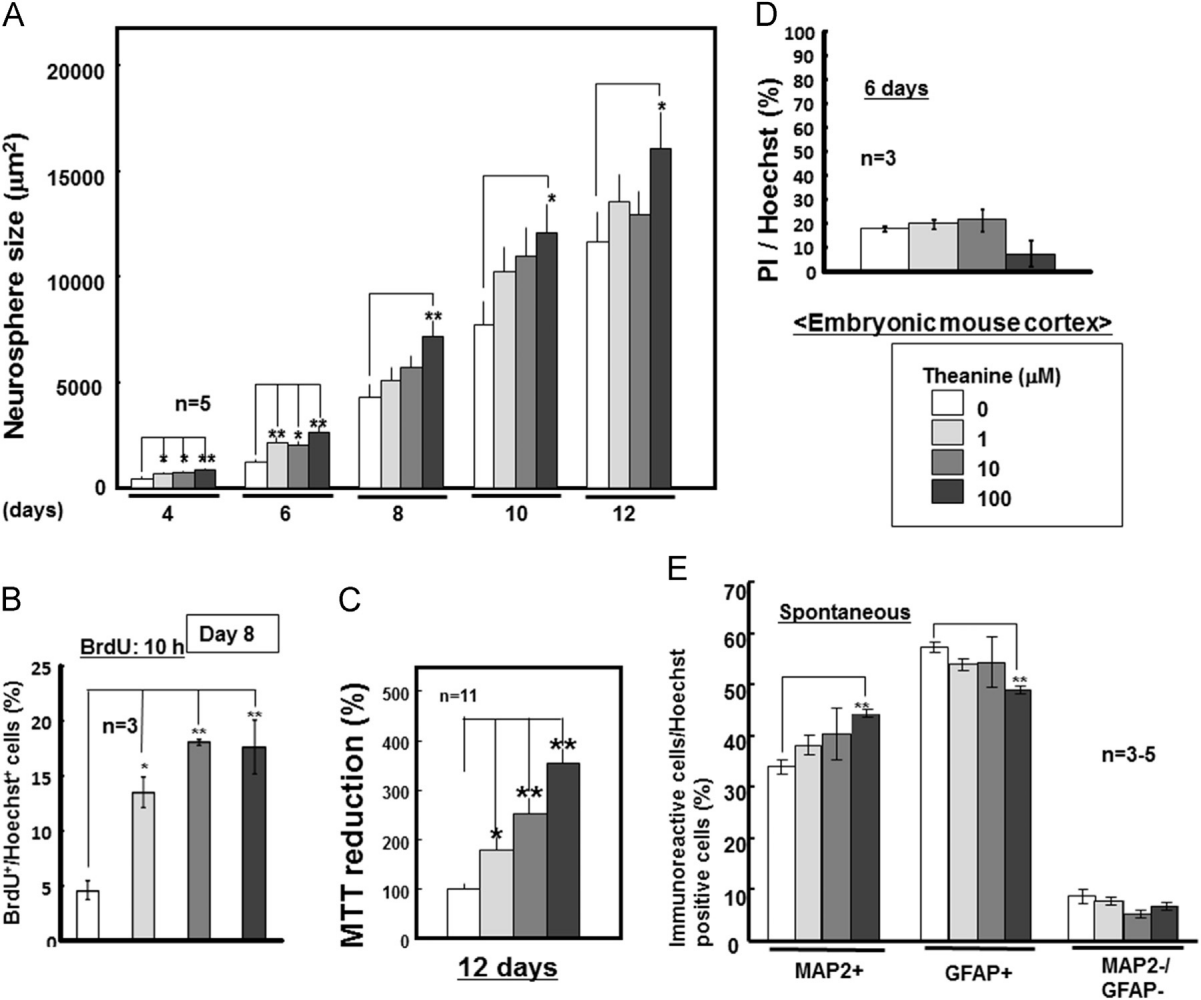
Effects of theanine on proliferation and differentiation in embryonic neural progenitors cells. Neural progenitor cells derived from embryonic mouse neocortex were cultured with EGF in either the presence or absence of theanine at different concentrations for 4 to 12 days, followed by measurement of (A) neurosphere size (*n*=5), (B) BrdU incorporation on Day 8 (*n*=3), (C) MTT reduction on Day 12 (*n=*11) and (D) double staining for DNA with Hoechst33342 and PI on Day 6 (*n*=3). (E) Cells were also cultured with EGF in either the presence or absence of theanine for 12 days, followed by removal of EGF and subsequent dispersion for further culture without theanine for an additional 6 days to induce spontaneous differentiation. The quantitative data show percentages of cells immunoreactive for either MAP2 or GFAP over the total number of cells stained with Hoechst33342 (*n*=3–5). *P<0.05, **P<0.01, significantly different from each control value obtained in cells not exposed to theanine. Statistical significance was determined using the one- or two-way ANOVA with Bonferroni/Dunnett *post hoc* test.

**Fig. 5:**
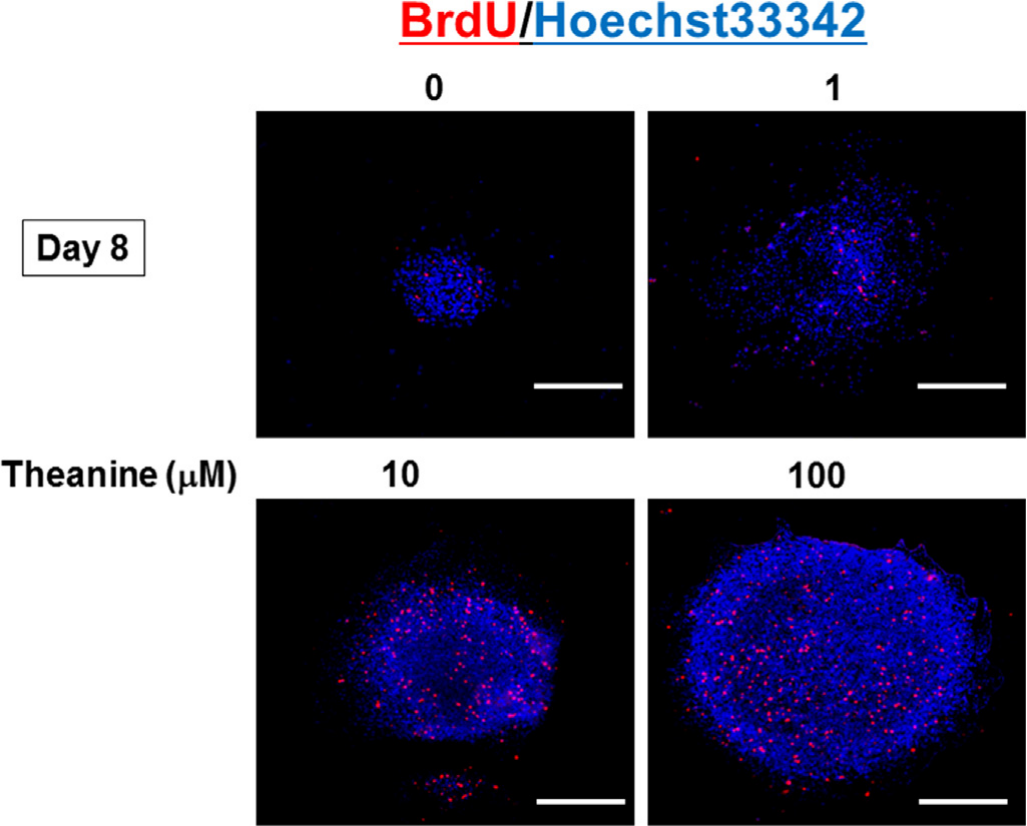
Neurospheres cultured for 8 days were incubated with 10 μM BrdU for 10 h, followed by immunocytochemical detection of BrdU incorporated along with Hoechst33342 staining.

We evaluated the effects of theanine on the proliferation activity in undifferentiated neural progenitor cells isolated from embryonic mouse neocortex *in vitro*. Sustained exposure to theanine led to a significant increase in the neurosphere size during culture for 12 days (Fig. 4A) and BrdU incorporation on Day 8 (Figs. 4B and 5), in addition to increasing MTT reducing activity on Day 12 (Fig. 4C), in a concentration-dependent manner at a concentration range of 1–100 μM. However, theanine did not significantly affect the ratio of cells stained with PI over those by Hoechst33342 as an index of cellular viability on Day 6 at concentrations used (Fig. 4D). To maximize the concentration-dependent pharmacological property, subsequent cellular differentiation was examined in neurospheres exposed to theanine at the highest concentration used. Prior culture with 100 μM theanine significantly increased the number of cells immunoreactive for MAP2 with a concomitant decrease in that for GFAP, whereas no significant change was seen in the number of cells not immunoreactive for either MAP2 or GFAP (Fig. 4E).

### 3.4 Adult mouse hippocampal progenitors

**Fig. 6:**
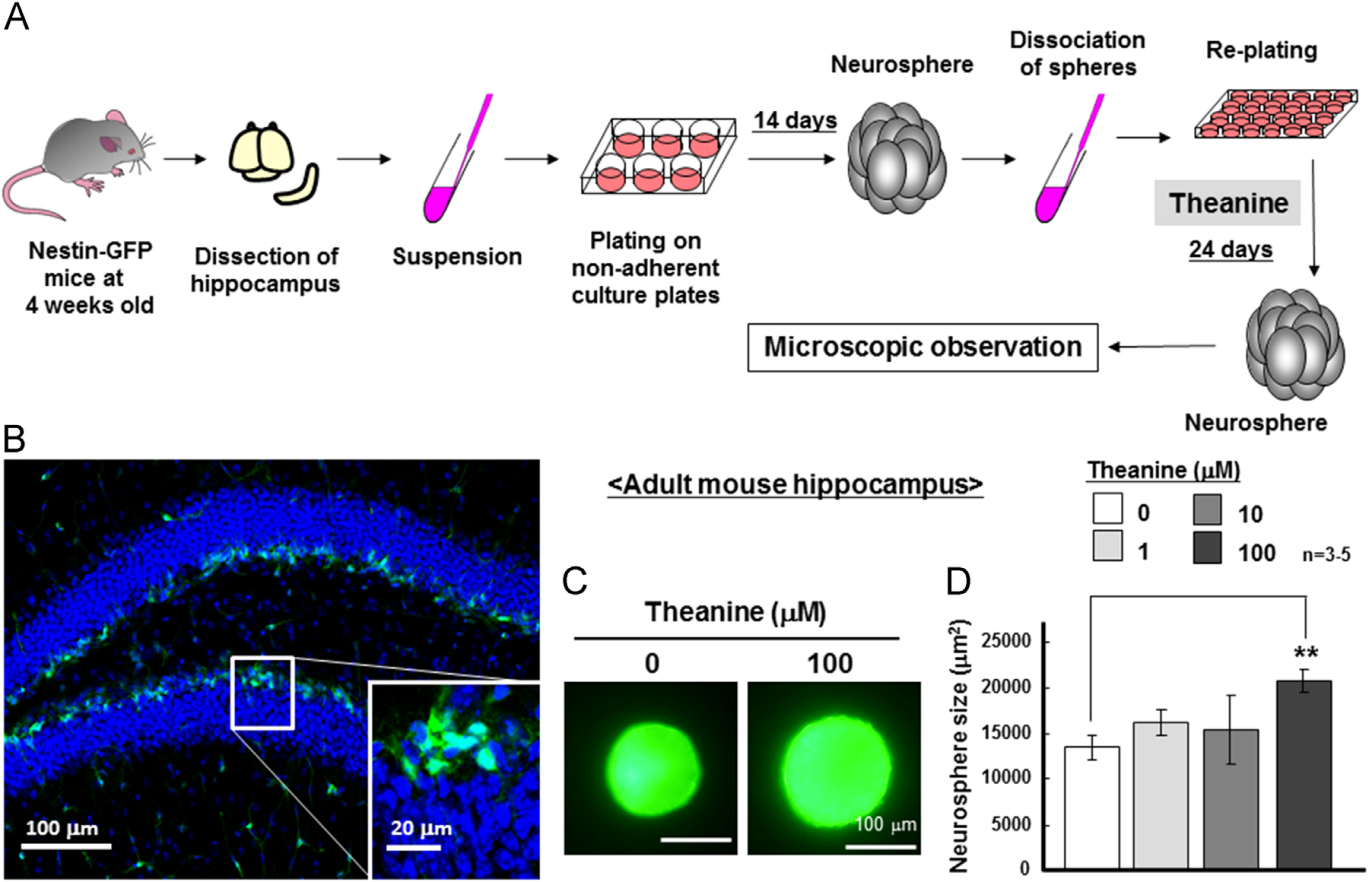
Effects of theanine on neurosphere size in adult hippocampal progenitor cells. Neural progenitor cells were isolated from the hippocampus of *Nestin*-GFP transgenic mice at 4 weeks old, followed by culture with both EGF and FGF for 14 days under floating conditions and subsequent dispersion for re-plating. Cells were then further cultured for an additional 24 days in either the presence or absence of theanine at different concentrations for measurement of the size of neurospheres formed as summarized in the panel (A). (B) *Nestin*-GFP transgenic mice at 4 weeks old were perfused with PA for dissection of hippocampal sections, followed by staining with Hoechst33343 and subsequent observation of the DG under fluorescence microscope. Hippocampal neurospheres from adult *Nestin*-GFP transgenic mice were cultured with theanine at different concentrations. Typical fluorescence pictures are shown in the panel (C), while the panel (D) shows quantitative analysis on the size of neurospheres formed. *P<0.05, significantly different from each control value obtained in cells not exposed to theanine. Statistical significance was determined using the one-way ANOVA with Bonferroni/Dunnett *post hoc* test.

To further elucidate pharmacological properties of theanine in adult neurogenesis, we employed neurospheres prepared from the hippocampus of young mice with predominant expression of GFP in cells expressing nestin [14]. Due to limited availability of neural progenitor cells from the hippocampus of adult *Nestin*-GFP transgenic mice, hippocampal neurospheres were formed during the initial culture for 14 days, followed by dispersion and subsequent further culture with theanine at concentrations of 1–100 μM for an additional 24 days (Fig. 6A). In hippocampal sections dissected from adult *Nestin*-GFP transgenic mice, GFP fluorescence was detected in the subgranular zone known to be enriched of neural progenitor cells expressing nestin below the granular cell layer stained with Hoechst33342 (Fig. 6B). Culture with 100 μM theanine markedly increased the size of neurospheres formed from the adult mouse hippocampus (Fig. 6C). Repetition of these experiments revealed a significantly increased size of adult mouse hippocampal neurospheres exposed to 100 μM theanine for 24 days (Fig. 6D).

## 4 Discussion

The essential importance of the present findings is that the green tea ingredient theanine, which is an amino acid with a structural analog to Gln rather than glutamate, promoted the phosphorylation of intracellular signaling molecules relevant to the mTOR kinase pathway in cultured neural progenitor cells isolated from embryonic mouse neocortex, in addition to facilitating both activities for proliferation and subsequent neuronal differentiation. The abundance of neural progenitor cells in the neocortex as well as the hippocampus is confirmed in embryonic rodent brains in our previous studies using embryonic brains of rats [17] and mice [12]. The fact that prolonged exposure to theanine increased the size of neurospheres obtained from the hippocampus of adult *Nestin*-GFP transgenic mice gives support for the proposal that theanine promotes proliferation for self-replication in adult neurogenesis. There was a clearly high positive correlation between the size of round neurospheres formed of clustered cells and BrdU incorporation as an index of the proliferation activity required for self-replication in neural progenitor cells [12], [17].

As [^3^H]theanine uptake was highly sensitive to the competitive inhibition by Gln in rat brain synaptosomes and *vice versa* [4], theanine seems to be a good substrate for several Gln transporters expressed in the brain. Glutamine was required for cell proliferation in a variety of cell types such as lymphocytes, enterocytes and tumor cells [18], while Gln facilitated proliferation through stimulation of nucleotide synthesis in Caco-2 cells [19]. Although membrane transportation of Gln was shown to regulate mTOR kinase activity, translation and autophagy for coordination of cell growth and proliferation [8], however, the exact mechanism by which theanine promoted the phosphorylation of key molecules required for mTOR signaling in place of Gln in undifferentiated neural progenitor cells is not clarified so far. Both theanine and Gln could be similarly incorporated into the cytoplasm through particular Gln transporters expressed on cellular surface, with differential profiles for activation of intracellular mTOR kinase signaling processes. One possible but hitherto unproven speculation is that theanine would be more efficient than Gln in facilitating the mTOR signaling pathway in neural progenitor cells in a particular situation. For example, Gln but not theanine is metabolized through glutaminolysis to produce α-ketoglutarate, which in turn promotes the intracellular signaling mediated by mTOR kinases in combination with the essential amino acid leucine [20]. Future comparative pharmacological profiling between theanine and Gln in undifferentiated neural progenitor cells undoubtedly gives a clue for elucidating molecular mechanisms underlying the activation by theanine of the mTOR signaling pathway.

In contrast, mTOR signals are essential for the maintenance of neural progenitor status in association with upregulation of several repressor basic helix-loop-helix (bHLH) genes, such as *Hes5*, in undifferentiated P19 cells [21]. Furthermore, Gln inhibited activation by arginine and leucine of the mTOR signaling in cultured rat intestinal epithelial cells [22]. In cultured Jarkat cells, mTOR activation was only seen in the presence of an extracellular amino acid complemented with Gln as an inevitable component [23]. Increased levels of intracellular Gln resulted in promotion of the influx of extracellular essential amino acids, such as leucine, in exchange for the efflux of intracellular Gln, which would then activate the mTOR signaling pathway required for protein translation, cell growth and reduced autophagy, toward facilitation of cell growth and proliferation [8]. In stable *Slc38a1* transfectant cells, marked upregulation was seen in *mRNA* expression of both activator (*Mash1, Math3, NeuroD1*) and repressor (*Hes5*) types of transcription factors with a bHLH domain [10]. These previous findings together with current findings, give rise to a speculative idea that sustained exposure to theanine could stimulate the phosphorylation mediated by mTOR signals of particular target proteins required for the induction of bHLH genes capable of modulating both proliferation and subsequent differentiation into particular lineages, which occurs in an autophagy-independent manner, in undifferentiated neural progenitor cells.

## Author contributions

Substantial contribution to conception and design, data acquisition, analysis or interpretation; T.T., N.N., R.N., T.K., H.K., S.I., S.N., N.K., E.H., Y.Y.

Drafting the article or revising it critically for important intellectual content; T.T., Y.Y.

Final approval of the version to be published; Y.Y.
