# Supplementary material for: Possible activation by the green tea amino acid theanine of mammalian target of rapamycin signaling in undifferentiated neural progenitor cells in vitro
Source: Biochem Biophys Rep. 2015 Dec 1;5:89–95. doi: 10.1016/j.bbrep.2015.09.021 (PMC5600317; doi:10.1016/j.bbrep.2015.09.021)
Supplement: Supplementary file 1 — Supporting material [file mmc1.docx]

**Conflict of interest**

All authors have no conflicts of interest to declare.
